# Vitamin D Supplementation and Treatment‐Free Survival in Early‐Stage CLL: A Real‐World Validation Study

**DOI:** 10.1002/jha2.70336

**Published:** 2026-07-02

**Authors:** Tamar Tadmor, Ronen Arbel, Talish Razi, Lior Rokach

**Affiliations:** ^1^ Hematology Unit Bnai Zion Medical Center and the Ruth and Bruce Rappaport Faculty of Medicine Technion Haifa Israel; ^2^ Community Medical Services Division Clalit Health Services Tel Aviv Israel; ^3^ Maximizing Health Outcomes Lab Sapir College Sderot Israel; ^4^ Department of Software and Information Systems Engineering Ben‐Gurion University of the Negev Beer‐Sheva Israel

**Keywords:** chronic lymphocytic leukemia, CLL, vitamin D

## Abstract

**Background:**

Patients with early‐stage chronic lymphocytic leukemia (CLL) are typically managed with a watch‐and‐wait strategy, and no intervention has been shown to modify the natural history of the disease.

**Methods:**

We analyzed a large real‐world cohort derived from Clalit Health Services, including patients diagnosed with early‐stage CLL between 2000 and 2022 who were initially managed with active surveillance. Exposure was defined as vitamin D supplementation for at least 6 months during the watch‐and‐wait period. The primary endpoint was treatment‐free survival (TFS). Multivariable time‐dependent Cox models and inverse probability of treatment weighting were applied to adjust for baseline differences.

**Results:**

The cohort included 5496 patients, of whom 1862 (34%) received vitamin D supplementation. Median age at diagnosis was 72.2 years, and 56% were male. Median follow‐up was 46 months. Patients receiving vitamin D demonstrated significantly longer TFS compared with nonusers (median 147 vs. 82 months, *p* <0.001). In multivariable time‐dependent Cox analysis adjusted for age, sex, and laboratory parameters, vitamin D exposure remained independently associated with improved TFS (HR: 0.87, 95% CI: 0.75–0.99; *p* = 0.027).

**Conclusions:**

In this large real‐world validation cohort, vitamin D supplementation was associated with prolonged TFS in patients with early‐stage CLL managed with active surveillance.

**Trial Registration:**

The authors have confirmed clinical trial registration is not needed for this submission.

## 1 Introduction

Chronic lymphocytic leukemia (CLL) is the most common leukemia in Western countries and is characterized by a highly heterogeneous clinical course [1]. To date, there is no indication to initiate therapy in patients with asymptomatic or low‐stage disease. Consequently, a watch‐and‐wait (W&W) strategy is frequently adopted at diagnosis. At this stage, management of patients with early‐stage CLL is primarily focuses on supportive care, as no intervention has been definitively shown to alter the natural history of the disease or prevent progression [1].

Regarding the association between vitamin D and CLL, previous studies have demonstrated that B‐CLL cells exhibit higher expression of the vitamin D receptor compared with normal B cells, suggesting a potential role for vitamin D in regulating key pathways involved in CLL proliferation and survival [2, 3]. In addition, vitamin D may play a role in the apoptotic regulation of B cells, as pharmacologic doses of vitamin D derivatives have been shown in vitro to induce apoptosis through activation of caspase 3‐ and 9‐dependent pathways [4, 5]. Another possible mechanism is the impact of low vitamin D levels on the tumor microenvironment, as vitamin D deficiency may enhance the activity of myeloid‐derived suppressor cells, which can promote leukemia cell growth through upregulation of regulatory molecules such as miR‐155 [3].

In our recent publication, we demonstrated that vitamin D supplementation in patients with early‐stage CLL managed with a W&W approach was associated with prolonged treatment‐free survival (TFS), as well as a longer time to first treatment (TTFT) in younger patients [6]. However, given the retrospective design and lack of prospective validation, confirmation in an independent cohort was warranted.

## 2 Methods

To address this, we analyzed a large real‐world cohort derived from Clalit Health Services (CHS), the largest healthcare provider in Israel, covering approximately 50% of the national population after receiving approval from the institution's ethical committee. The study period spanned from January 1, 2000, to December 31, 2022. Using the ICD‐9 coding system and electronic medical records, we identified 5505 patients diagnosed with CLL who met iwCLL diagnosis criteria and were initially managed with a W&W approach for at least 3 months. The median age at diagnosis was 72.2 years, and 55.6% were male.

Patients were followed from diagnosis until initiation of the first CLL‐directed therapy or censoring. Exposure to vitamin D was defined as receipt of vitamin D supplementation, a vitamin D analog, or both, for at least 6 months during the W&W period. The cohort was divided into two groups: Patients who received vitamin D and/or a vitamin D analog and those who did not. The study was conducted in accordance with the Declaration of Helsinki and was approved by the Institutional Review Board/Ethics Committee of CHS.

The primary endpoints were TFS and TTFT. TFS was defined as a composite endpoint of TTFT or death, whereas in the TTFT analysis, death was treated as a censoring event.

We employed a multivariable time‐dependent Cox proportional hazards model adjusted for age and sex to account for variations in vitamin D exposure during follow‐up. To further reduce confounding, we used inverse probability of treatment weighting (IPTW) with estimation of the average treatment effect on the treated (ATT), thereby creating a pseudo‐population in which baseline covariates were balanced between exposed and unexposed groups. While both propensity score matching and IPTW can mitigate bias when estimating marginal hazard ratios, IPTW was selected due to evidence suggesting greater statistical precision. The IPTW procedure consisted of two steps. First, we calculated propensity scores representing the probability of exposure to vitamin D based on baseline characteristics (age, sex, white blood cell count, platelet count, and hemoglobin level). Second, we derived weights as the inverse of the propensity score and fitted a weighted Cox proportional hazards model. Laboratory parameters were included as time‐varying covariates. Given that blood tests were obtained at irregular intervals, missing intermediate values were imputed using linear interpolation. Other time‐varying variables (e.g., vitamin D supplementation status) were not imputed. Multicollinearity among covariates was assessed using the variance inflation factor (VIF). The proportional hazards assumption was evaluated using Schoenfeld's global test. The Mantel–Byar test was applied to address immortal time bias. All statistical analyses were performed using R version 4.2.2 (October 31, 2022) (R Foundation for Statistical Computing, Vienna, Austria).

## 3 Results and Discussion

Among 5496 patients included in the final analysis, 1862 received vitamin D supplementation and/or vitamin D analogs, and 3634 did not. The median follow‐up duration from diagnosis to first treatment or death was 46 months (19–89). Additional baseline clinical and laboratory characteristics are summarized in Table 1.

**TABLE 1:** Baseline characteristics of patients with CLL by vitamin D exposure status.

Variable	Total cohort (*N* = 5496)	Non–vitamin D users (*N* = 3634)	Vitamin D users (*N* = 1862)	*p* value
Age at diagnosis, median (IQR)	72.2 (63.6–79.8)	71.1 (62.2–79.1)	74.1 (66.6–80.8)	<0.001
Male sex, *n* (%)	3062 (56%)	2380 (65%)	682 (37%)	<0.001
Binet stage A∖B, *n* (%)	5184 (94%)	3452 (95%)	1732 (93%)	0.081
Hemoglobin (g/dL), median (IQR)	13.1 (11.9–14.2)	13.2 (11.8–14.3)	12.9 (12.0–13.9)	<0.001
Platelets (×10^9^/L), median (IQR)	196.0 (154.0–244.0)	192.0 (151.0–243.0)	202.0 (160.0–248.0)	<0.001
WBC (×10^9^/L), median (IQR)	19.9 (14.3–31.9)	20.5 (14.5–33.5)	18.7 (13.9–29.2)	<0.001
Absolute lymphocyte count, median (IQR)	12.6 (8.0–23.0)	13.1 (8.1–23.9)	12.0 (7.8–21.0)	<0.001
Vitamin D level at baseline (ng/mL), median (IQR)	26.91 (20.17–33.60)	26.96 (20.17–34.26) NA: 92%	25.95 (19.75–33.24) NA: 76%	0.315
Follow‐up time, months, median (IQR)	46 (19–89)	37 (14–75)	68 (34–113)	<0.001

**FIGURE 1:**
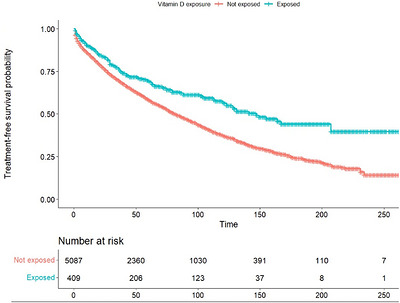
Expected treatment‐free survival after adjustment to age, sex, and baseline laboratory parameters for comparing two groups: non–vitamin D users; vitamin D supplement users.

Patients receiving vitamin D demonstrated significantly longer TFS compared with nonusers (median TFS: 147 vs. 82 months; *p* <0.001; Figure 1). This association remained significant after adjustment for age, sex, and baseline laboratory parameters and considering of 3 months lag exposure (see Table S1). No statistically significant difference was observed in TTFT between the groups (*p* = 0.14).

Consistent with the prior MHS cohort [6], vitamin D users in the CHS cohort were older (median age 74.1 vs. 71.1 years), more likely to be female (63% vs. 35%), and had more favorable hematologic parameters, including higher platelet counts and lower WBC. Baseline vitamin D serum levels were similar between users and nonusers in both cohorts (CHS: *p* = 0.315; MHS: *p* = 0.294), underscoring that supplementation practice was not driven by documented deficiency.

When comparing our previous results based on Maccabi Health Service (MHS): The median follow‐up from diagnosis to first treatment or death was 46 months (IQR: 19–89) in the CHS cohort versus 57 months (IQR: 20–122) in the MHS cohort [6]. Patients receiving vitamin D demonstrated significantly longer TFS compared with nonusers in both cohorts (CHS: 147 vs. 82 months; MHS: 169 vs. 84 months; both *p* ≤ 0.004). This association remained significant after multivariable adjustment incorporating a 3‐month lag for vitamin D exposure in CHS (HR: 0.866, 95% CI: 0.751–0.989; *p* = 0.027) and was similarly significant in MHS (HR: 0.912, 95% CI: 0.856–0.971; *p* = 0.004; Table S1). No statistically significant difference in TTFT was observed in the CHS cohort (*p* = 0.14).

Several previous small cohort studies in patients with CLL have evaluated vitamin D insufficiency and reported that low vitamin D levels are associated with shorter TTFT and inferior overall survival [7, 8, 9, 10]. Our previous study demonstrated that administration of vitamin D or its non‐endogenous analog to patients with CLL in a W&W active surveillance is significantly associated with a longer TFS. In the present independent validation cohort, we extend these observations by demonstrating that vitamin D supplementation is associated with improved TFS.

Sex‐related differences in vitamin D use should also be considered when interpreting our findings. Although CLL is more prevalent in men, vitamin D supplementation in the present cohort was substantially more common among women. A similar pattern was observed in our previously published cohort, suggesting that this imbalance likely reflects general population health behavior rather than disease‐related factors, as women are more frequently prescribed or self‐administer vitamin D supplements. Consequently, the vitamin D‐exposed group included a higher proportion of women, who may differ from men in health‐seeking behavior, comorbidity burden, and healthcare utilization. Although our multivariable models adjusted for sex, residual confounding related to differential supplement use cannot be fully excluded and may have introduced bias against men in the non–vitamin D group.

A limitation of this study is the lack of prospective trials demonstrating a causal benefit of vitamin D supplementation in CLL. In addition, a recent prospective study in indolent lymphomas treated with rituximab did not show benefit from routine vitamin D supplementation [11]. Yet, these findings cannot be directly extrapolated to CLL due to the distinct biological characteristics of CLL cells.

This represents the second retrospective study led by our group examining the association between vitamin D and clinical outcomes in early‐stage CLL. The consistency of findings across two independent real‐world cohorts strengthens the biological plausibility and clinical relevance of this association. Although residual confounding cannot be excluded, the reproducibility of the results supports the hypothesis that vitamin D supplementation may be associated with a more indolent disease course in patients with early‐stage CLL managed with active surveillance.

Importantly, the consistency of this pattern across two independent real‐world cohorts supports the robustness of the observed association.

A prospective randomized study is unlikely to be feasible for several reasons: First, vitamin D insufficiency is highly prevalent in the general population. In addition, postmenopausal women commonly receive vitamin D supplementation as part of routine clinical care, making it difficult from ethical and medical prospective to justify withholding supplementation in this population. In this context, the current validation cohort provides novel support for the potential beneficial effect of vitamin D supplementation during active surveillance.

## Author Contributions

T.T. and L.R. designed and wrote the manuscript. L.R., T.R., and R.A. provided the data and performed the analysis.

## Funding

The authors have nothing to report.

## Conflicts of Interest

The authors declare no conflicts of interest.

## Supporting information



Supporting Information: jha270336‐sup‐0001‐SuppMat.docx

## Data Availability

The data that support the findings of this study are available on request from the corresponding author. The data are not publicly available due to privacy or ethical restrictions.

## References

[1] M. Hallek , B. D. Cheson , D. Catovsky , et al., “iwCLL Guidelines for Diagnosis, Indications for Treatment, Response Assessment, and Supportive Management of CLL,” Blood 131, no. 25 (2018): 2745–2760, 10.1182/blood-2017-09-806398. 29540348

[2] M. Gerousi , F. Psomopoulos , K. Kotta , et al., “The Calcitriol/Vitamin D Receptor System Regulates Key Immune Signaling Pathways in Chronic Lymphocytic Leukemia,” Cancers 13 (2021): 285. 33466695 10.3390/cancers13020285 PMC7828837

[3] H. Bruns , M. Böttcher , M. Qorraj , et al., “CLL‐Cell‐Mediated MDSC Induction by Exosomal miR‐155 Transfer Is Disrupted by Vitamin D,” Leukemia 31, no. 4 (2017): 985–988, 10.1038/leu.2016.378. 28008175

[4] C. T. A. Pepper , T. Hoy , D. Milligan , P. Bentley , and C. Fegan , “The Vitamin D3 Analog EB1089 Induces Apoptosis via a p53‐Independent Mechanism in Volving p38 MAP Kinase Activation and Suppression of ERK Activity in B‐Cell Chronic Lymphocytic Leukemia Cells In Vitro,” Blood 101, no. 7 (2003): 2454–2459, 10.1182/blood-2002-07-1984. 12446453

[5] J.‐B. Arlet , C. Callens O. Hermine , et al., “Chronic Lymphocytic Leukaemia Responsive to Vitamin D Administration,” British Journal of Haematology 156 (2011): 129–152. 21883139 10.1111/j.1365-2141.2011.08828.x

[6] T. Tadmor , G. Melamed , H. Alapi , S. Gazit , T. Patalon , and L. Rokach , “Vitamin D Supplement for Patients With Early‐Stage Chronic Lymphocytic Leukemia Is Associated With a Longer Time to First Treatment,” Blood Advances 8, no. 14 (2024): 3840–3846, 10.1182/bloodadvances.2023011458. 38701347 PMC11318322

[7] S. Molica , G. Digiesi , A. Antenucci , et al., “Vitamin D Insufficiency Predicts Time to First Treatment (TFT) in Early Chronic Lymphocytic Leukemia (CLL),” Leukemia Research 36, no. 4 (2012): 443–447, 10.1016/j.leukres.2011.10.004. 22047708

[8] J. L. Kelly , G. Salles , B. Goldman , et al., “Low Serum Vitamin D Levels Are Associated With Inferior Survival in Follicular Lymphoma: A Prospective Evaluation in SWOG and LYSA Studies,” Journal of Clinical Oncology 33, no. 13 (2015): 1482–1490, 10.1200/JCO.2014.57.5092. 25823738 PMC4404425

[9] J. G. Sfeir , M. T. Drake , B. R. LaPlant , et al., “Validation of a Vitamin D Replacement Strategy in Vitamin D‐Insufficient Patients With Lymphoma or Chronic Lymphocytic Leukemia,” Blood Cancer Journal 7 (2017): e526, 10.1038/bcj.2017.9. 28157213 PMC5386343

[10] S. A. Parikh and T. D. Shanafelt , “Vitamin D Insufficiency in CLL: A Modifiable Prognostic Factor?,” Blood Advances 8, no. 14 (2024): 3838–3839, 10.1182/bloodadvances.2024013428. 39042381 PMC11369631

[11] J. W. Friedberg , M. T. Brady , M. Strawderman , et al., “Vitamin D in Patients With Low Tumor‐Burden Indolent Non‐Hodgkin Lymphoma Treated With Rituximab Therapy (ILyAD): A Randomized, Phase 3 Clinical Trial,” EClinicalMedicine 78 (2024): 102959, 10.1016/j.eclinm.2024.102959. 39677358 PMC11638608

