# Supplementary material for: Vitamin D Supplementation and Treatment‐Free Survival in Early‐Stage CLL: A Real‐World Validation Study
Source: EJHaem. 2026 Jul 2;7(4):e70336. doi: 10.1002/jha2.70336 (PMC13325541; doi:10.1002/jha2.70336)
Supplement: Supplementary file 1 — Supporting Information: jha270336‐sup‐0001‐SuppMat.docx [file JHA2-7-e70336-s001.docx]

Supplement table 1: Multivariable Cox regression with time-dependent covariates for TFS

| Variable | HR | p-value |
| --- | --- | --- |
| Age during Diag. | 1.0496 (1.0469-1.0551) | <0.001 |
| Sex: male | 1.1258 (1.0459-1.2107) | 0.0014 |
| Vitamin D (3 months lag) | 0.8656 (0.7507-0.9893) | 0.0273 |
| WBC (×10⁹/L) | 1.003 (1.002-1.003) | <0.001 |
| Platelet (×10⁹/L) | 0.997 (0.996-0.999) | <0.001 |
| Hemoglobin Gr/dL | 0.845 (0.821-0.868) | <0.001 |
